# Different hemoglobin thresholds for transfusion in patients with severe sepsis and septic shock

**DOI:** 10.1186/2197-425X-3-S1-A89

**Published:** 2015-10-01

**Authors:** T Kamio, M Sanui, M Hayakawa, D Kudo, K Yamakawa, Y Iizuka, S Saito, K Takimoto, S Uchino, T Mayumi, Y Sasabuchi, Japan-SEPTIC DIC Study Group

**Affiliations:** Department of Anesthesiology and Critical Care Medicine, Jichi Medical University Saitama Medical Center, Saitama, Japan; Hokkaido University Hospital, Emergency and Critical Care center, Sapporo, Japan; Division of Emergency and Critical Care Medicine, Tohoku University Graduate School of Medicine, Sendai, Japan; Division of Molecular & Vascular Medicine, Beth Israel Deaconess Medical Center, Boston, United States; Intensive Care Unit, Department of Anesthesiology, Jikei University School of Medicine, Tokyo, Japan; Department of Anesthesiology and Intensive Care Medicine, Osaka University, Osaka, Japan; Department of Emergency Medicine, University of Occupational and Environmental Health, Kitakyushu, Japan; Department of Clinical Epidemiology and Health Economics, School of Public Health, University of Tokyo, Tokyo, Japan

## Introduction

Hemoglobin (Hb) level transfusion thresholds in patients with sepsis who develop anemia have long been a matter of debate. In a randomized controlled trial comparing thresholds of Hb ≤ 7 g/dL vs. ≤ 9 g/dL in patients with severe sepsis and septic shock, comparable outcomes including mortality were obtained [1], while in an observational study of those patients, higher Hb levels were associated with lower mortality [2]. During the resuscitation phase of septic shock, a substantial number of clinicians may have used a higher threshold of hemoglobin at 10 g/dL, as recommended in the Surviving Sepsis Campaign Guideline 2008 [3].

## Objectives

In this retrospective study of patients with severe sepsis and septic shock, we investigated whether a higher transfusion threshold of Hb≤11g/dL or higher, compared with Hb≤9g/dL, was associated with improved outcomes including ICU and in-hospital mortality.

## Methods

This is a retrospective study from the database of the J-SEPTIC DIC study conducted in 41 ICUs, which was developed to evaluate an association between sepsis-related coagulopathy, anticoagulation therapies, and clinical outcomes in 3195 adult patients with severe sepsis and septic shock admitted to ICUs in Japan from January 2011 through December 2013. Patients were divided into three groups with virtual transfusion thresholds according to the lowest hemoglobin levels recorded during the first seven days of ICU stay: i) a threshold of Hb≤9g/dL, ii) Hb≤11g/dL, and iii) Hb>11g/dL. Patients either not transfused with the lowest Hb≤9g/dL or transfused with the lowest Hb>13 g/dL were excluded. Patients transfused with the lowest Hb levels ≤9g/dL or not transfused due to Hb levels spontaneously maintained >9g/dL and ≤ 11g/dL were assigned to the group with a threshold of Hb≤9g/dL. Equivalent assignment criteria were applied to the remaining two groups. To determine an association between RBC transfusion thresholds and mortality, multivariate logistic regression analysis was performed.

## Results

Of 3195 patients, 1423 patients (44%) received one or more RBC units during the first seven days of ICU stay. Crude ICU mortality in the Hb≤9g/dL, Hb≤ 11g/dL, and Hb>11 g/dL groups was 10.9, 7.3 and 7.5%, respectively (p= 0.074), while in-hospital mortality in each group was 26.4, 18.2 and 9.4%, respectively (p < 0.001). Using multivariate analysis, higher thresholds of Hb was not associated with ICU mortality (odds ratios: OR [confidence interval: CI] 1.19 [0.76-1.87] and 2.37 [0.75-7.53] for Hb≤11g/dL and Hb>11g/dL groups, respectively, compared with Hb≤9g/dL) or in-hospital mortality (OR 1.11 [0.75-1.66] and 0.94 [0.35-2.56], respectively).

## Conclusions

In this retrospective multicenter observational study of patients with severe sepsis and septic shock, transfusion thresholds with a Hb>9g/dL were not associated with reduced mortality.
